# Use of Micro-CT Imaging to Assess Ventral Mandibular Cortical Thickness and Volume in an Experimental Rodent Model With Chronic High-Phosphorus Intake

**DOI:** 10.3389/fvets.2021.759093

**Published:** 2021-12-09

**Authors:** Vladimir Jekl, Adam Brinek, Tomas Zikmund, Edita Jeklova, Josef Kaiser

**Affiliations:** ^1^Department of Pharmacology and Pharmacy, Faculty of Veterinary Medicine, Veterinary University Brno, Brno, Czechia; ^2^Jekl & Hauptman Veterinary Clinic, Brno, Czechia; ^3^CEITEC – Central European Institute of Technology, Brno University of Technology, Brno, Czechia; ^4^Department of Infectious Diseases and Preventive Medicine, Veterinary Research Institute, v.v.i., Brno, Czechia

**Keywords:** micro-CT, rodent, volume thickness, cortical bone, degu (*Octodon degus*), dentistry, dental disease

## Abstract

Adverse effects of high dietary phosphorus on bone health have been observed in both animal and human studies. The aim of the investigation was to examine chronic effects of high phosphorus diet on the apical mandibular cortical thickness and volume in a hystricomorph rodent (*Octodon degus*) using microcomputed tomography. Male degus were randomly divided into two groups fed by different mineral contents from the age of 12 weeks till the age of 17 months. The micro-CT scanning and wall thickness analysis were applied on the region of the mandible exactly under the apices of the 4th premolar tooth, first molar tooth, and second molar tooth in two animals from each group. General overview and mapping of the ventral mandibular bone thickness revealed pronounced bony mandibular protrusions in all the animals fed a high-phosphorus diet with obvious bone thinning apically to the 4th premolar and first and second molar tooth apices. Mandibular bone volume and thickness located apically to the premolar and molars were statistically significantly smaller/thinner in the group fed by a high phosphorus diet. The thinnest bone measured 0.004 mm, where the mandibular 4th premolar tooth almost perforated the mandibular cortex. Similar studies of metabolic bone disease and its influence on alveolar bone were also published in rats and mice. The influence of different environmental, infectious, or metabolic factors on the growing tooth, alveolar bone formation, and bone pathologies must be done experimentally on growing animals. In contrast, degus have continuously growing dentition, and the effect of any of the above listed factors can be studied in this animal model at any age and for longer time periods.

## Introduction

Metabolic bone diseases are a diverse group of diseases that result in abnormalities of bone mass, mineral homeostasis, bone turnover, or growth in man and also in animals (1). Apart from osteoporosis, the most commonly encountered metabolic bone diseases in humans are associated with impaired metabolism of phosphorus (2).

Phosphorus is widespread in human food supply in both natural organic forms and added inorganic forms, and humans have a high efficiency for dietary phosphorus absorption. Therefore, phosphorus deficiencies are rare. Instead, excessive dietary phosphorus intake is observed in nearly all age groups in the US (1, 3, 4). Adverse effects of high dietary phosphorus on bone health have been observed in both animal and human studies. A high-phosphorus diet produces a higher level of plasma phosphate, which reduces urine calcium loss, reduces renal synthesis of 1.25-dihydroxycholecalciferol, reduces serum-ionized calcium, and leads to increases in parathormone and osteopontin release with subsequent secondary hyperparathyroidism and increased bone resorption (1, 2, 5, 6).

The condition of the bone is reflected in the alveolar bone and erupting teeth. In man, because of the fast bone metabolism in the mandibular alveolar process, osteopenia and an increased skeletal fracture risk may first be detected here (7). Defective alveolar bone led to the pathological orthodontic tooth movement and increased risk of periodontal disease not only in humans but also in animals (8–11). High dietary phosphorus also increases the risk cariogenesis (12).

The use of degus (*Octodon degus, Octodontidae, Rodentia*), hystricomorphic medium-sized diurnal rodents, as a laboratory subject has grown over the last few decades due to their unique biological features, especially in the research of anatomy and physiology, aging, diabetes mellitus, vision, behavior, and social interactions (13–16). Studies published in privately kept degus have shown a very high incidence of spontaneous dental disease, which was characterized by apical and coronal clinical crown elongation, tooth sharp spike formation, and orthodontic tooth movement within the jaw (17). Experimental studies in degus showed that chronic high dietary phosphorus intake with adequate calcium and vitamin D intake had deteriorative effects on degus dentition and cause enamel hypoplasia and mandibular osteoporosis (18, 19). Disturbed mineral metabolism resulted in incisor enamel depigmentation, enamel hypoplasia, enamel pitting, and altered dentin morphology.

The aim of the present investigation was to examine the chronic effect of high-phosphorus diet on the mandibular apical alveolar bone thickness in a hystricomorph rodent—*Octodon degu*—using microcomputed tomography.

## Materials and Methods

### Animals, Diet, and Housing

Male degus (*Octodon degu*) were housed in three-level plastic and wire-mesh cages in the animal care facility with controlled conditions (day light 12/12 h, temperature 20–23°C, humidity 41–51%). A total of 28 animals were randomly divided into two groups fed by different mineral contents from the age of 12 weeks. Group A was fed with 13.2 g/kg calcium of dry matter basis and with 6.3 g/kg phosphorus of dry matter basis (calcium to phosphorus ratio 2.1:1). Group B was fed with 9.1 g/kg calcium of dry matter basis and with 9.5 g/kg phosphorus of dry matter basis (calcium to phosphorus ratio 0.96:1). The degus were fed twice a day by complete pelleted diet and had free access to water. Nutritional content (200 g/kg of dry matter basis, basal energy 19.1 MJ/kg), fiber (174 g/kg), and vitamin D level (697 UI/kg) were similar in both types of diets. The experiment complied with ARRIVE guidelines (20). The animals were housed and handled, and the experiment was performed with the agreement of the Institutional Animal Welfare Committee and Branch Commission for Animal Welfare of the Ministry of Agriculture of the Czech Republic (No. 46613/2003-1020). The animals were clinically examined regularly and euthanized at the age of 17 months.

### Macroscopic Observation

Degus were clinically examined on a weekly basis until the age of 20 weeks (8 weeks of feeding experimental diet) and then in 4-week intervals till the end of the experiment, until the age of 17 months. The macroscopic observation comprised of general health assessment (behavior, body condition score) and clinical examination—incisor evaluation, intraoral examination, and jaw palpation included.

### Radiography

Isolated lateral radiographs of mandibles were taken in all the animals post mortally.

### Micro-CT Scanning, Ventral Mandibular Cortical Thickness, and Volume

For the purpose of this study, two animals from each group were randomly selected and micro-CT of the mandibles was performed to compare the thickness of the ventral mandibular cortical thickness. Other animals used in experiment were used for the histopathological evaluation of internal organs (unpublished data) and for the incisor pathology assessment using electron and scanning microscopy (19). Computed tomography using a Somatom Emotion multislice scanner (Siemens AG, Germany) was used intravitally four times during the study on another five animals from each group (21). The micro-CT scanning was performed using laboratory system GE Phoenix v|tome|x L 240 equipped with a nano-focus X-ray tube with a 180-kV/15-W and high-contrast DXR250 flat panel with 2,048 × 2,048 pixel^2^. The exposure time was 900 ms in every 2,200 positions. In all measurements, the utilized acceleration voltage and X-ray tube current were 90 kV and 90 A, respectively. The beam was filtered by 0.5 mm of the copper filter. The voxel size of the obtained volume was 8 μm. The tomographic reconstruction was realized using the software GE phoenix datos|x 2.0 (GE Sensing and Inspection Technologies GmbH, Germany) with a sample drift and beam hardening correction. The data analysis was done in software VGStudio MAX 2.2 (Volume Graphics GmbH, Germany), including a thickness analysis module. The general 3D view of surface thickness was visualized with GOM Inspect V7.5.

**Figure 1:**
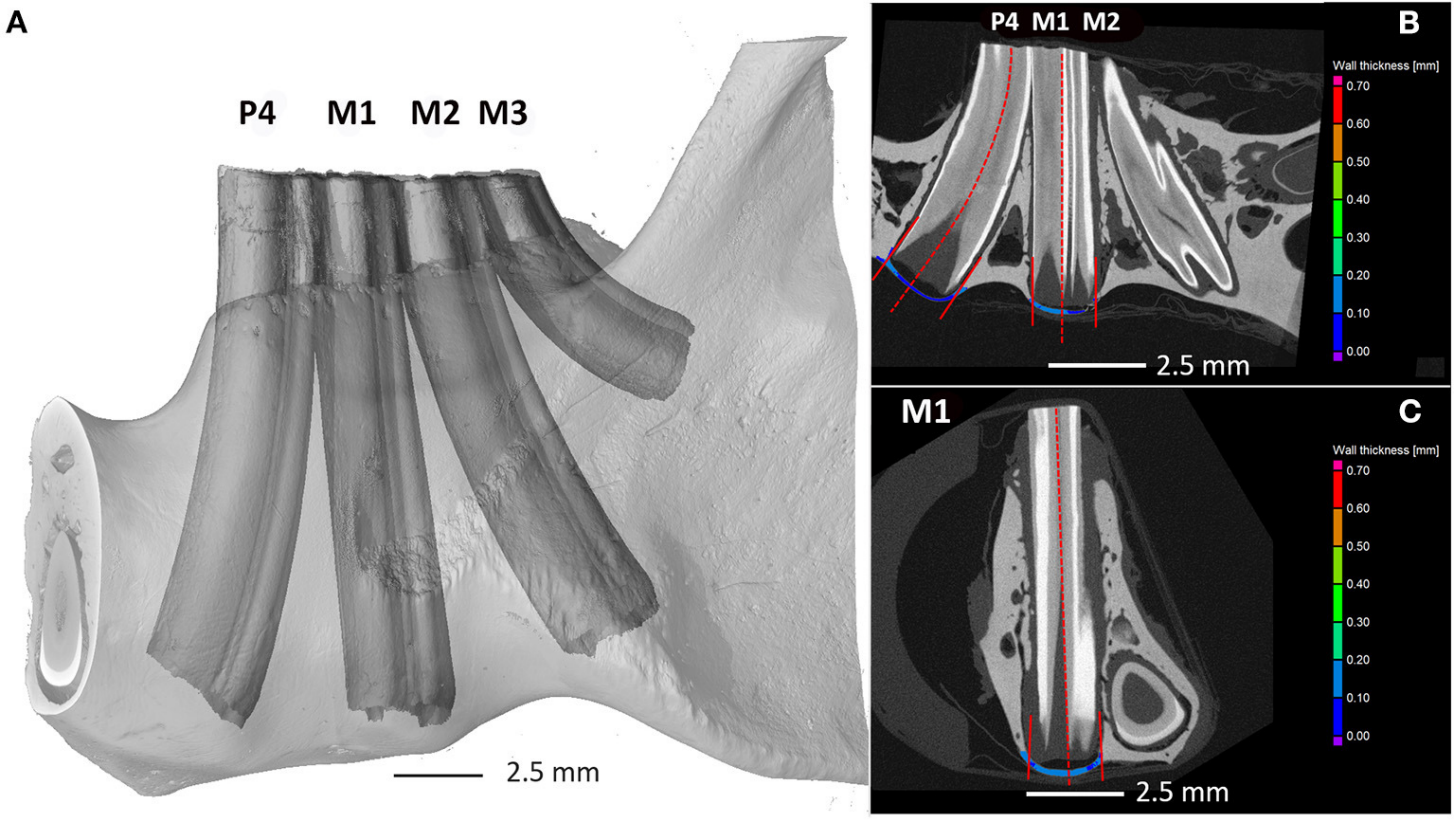
Degu mandible. Quantifying the mandibular bone thickness at the area of apical tooth elongation using the first method. **(A)** Three-dimensional render of scanned jaw with tooth labeling, **(B)** sagittal, and **(C)** coronal cross section showing the regions under the tooth apex analyzed by wall thickness analysis. P4—4th premolar tooth; M1—1st molar tooth; M2—2nd molar tooth.

At first, the segmentation of bone and tooth was done automatically by the VGStudio surface determination module. Then, the image analysis specialist created the regions to calculate the ventral mandibular cortical thickness and periodontal space. The borders of the bony area examined were defined as the projection of the hard substance (dentin, cementum, enamel) to the mandibular bone ventral to the apex. The wall thickness analysis module was applied on the region of the mandible strictly under the tooth apices of the 4th premolar tooth (A), first molar tooth (B), and second molar tooth (C) (Figure 1). The third molar was not included in the measurements as the curvature of the tooth did not allow proper measurements. The module calculates the thickness automatically and measures it as the smallest distance between the particular parts of the surface [from 0.000249 mm to 1.0 mm at a 0.000050 mm (50 μm)].

### Statistical Analysis

The data of particular volumes were tested by the Pearson normality test. Bone volumes and thickness of the mandibular bone apically to the particular tooth (P4, M1, or M2) and all the volumes (P4 to M1) were compared between two degu groups using the Mann–Whitney non-parametric test. The significance threshold was estimated as *p* ≤ 0.05.

## Results

### Animals and Macroscopic Observation

In group A, all animals behave normally and were in good body condition (body condition score (BCS, 5-6/10). The incisor enamel was yellowish to orange pigmented and was smooth in all animals. Ventral parts of both mandibles were smooth on palpation. Oral cavity examination at the age of 17 months did not reveal dental disease, i.e., clinical crowns of all the 4th premolar teeth and molar teeth were of the same size, growing in straight directions, and did not show any abnormalities.

In group B, all animals were in bad body condition (BCS 1-3/10). The incisor enamel was depigmented and had irregular rough surface, clinical crowns of the mandibular incisors were elongated, and maxillary incisors were of smaller height. Ventral parts of both mandibles were irregular with the presence of small bony swellings. In case of 4th premolar and molar teeth, oral cavity examination at the age of 17 months revealed severe dental disease, i.e., irregular occlusal surface of all the teeth, clinical crown elongation, different growth tooth shapes, wider diastemas, presence of enamel and dentin defects, and presence of soft tissue erosions.

### Radiography

Dental radiographs of the left mandible confirmed, in particular, a reserve crown elongation of all teeth in both the coronal and apical directions. Significant elongation of the mandibular incisors, 4th premolar, and all molar teeth were recorded in group B (17).

### CT Measurements

#### Quantifying the Ventral Mandibular Bone Thickness and Volume

**Figure 2:**
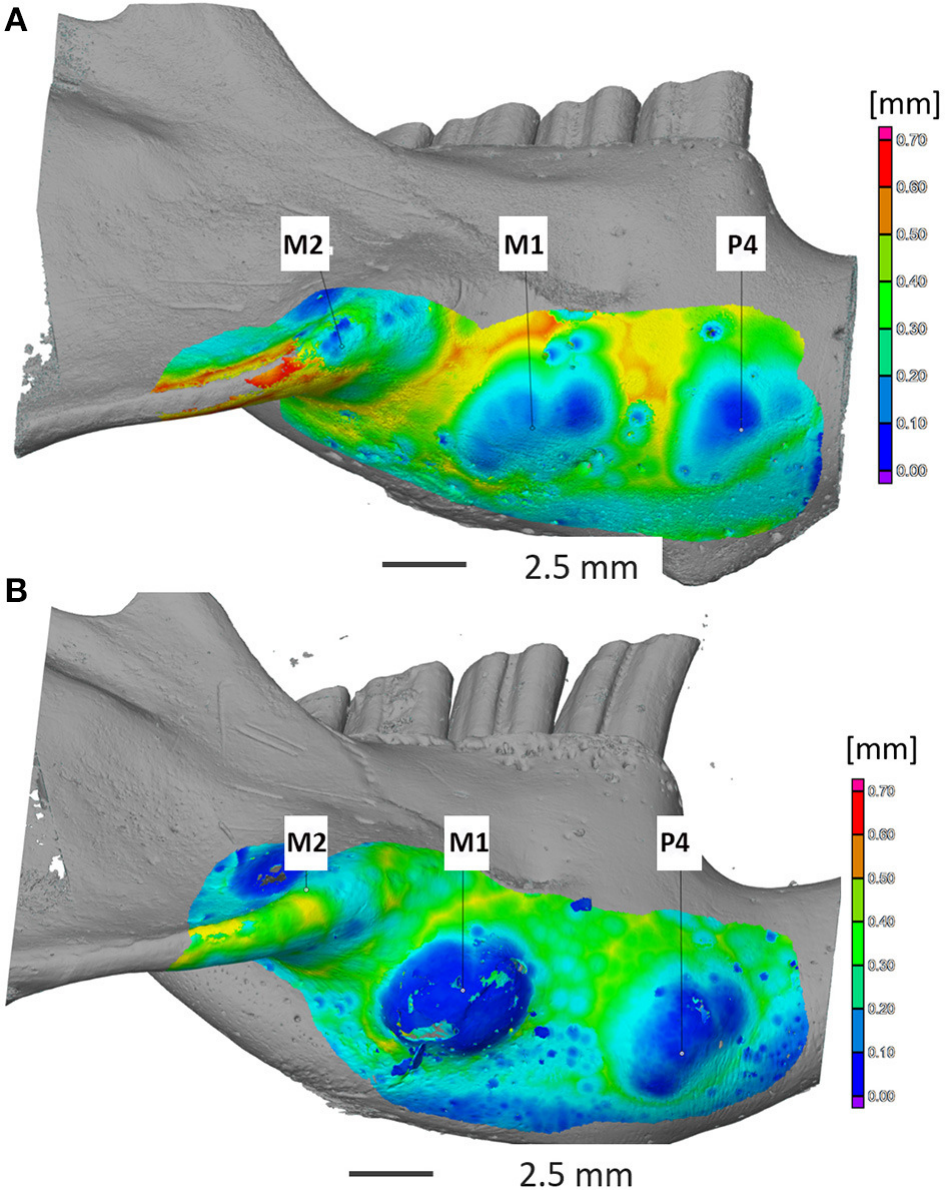
Computed tomography imaging of the mandibular cortical bone thickness at the apical area of the mandibular 4th premolar tooth and mandibular molars teeth in degus fed by normal **(A)** and high-phosphorus diets **(B)**. Note: severe thinning of the cortical bone ventral to the apex of P4 and M1 **(B)**. Apical elongation of mandibular 4th premolar and molar teeth was also palpable as bony swellings on the ventral mandibular surface **(B)**. Legend: P4—4th premolar tooth; M1—1st molar tooth; M2—2nd molar tooth.

General overview and mapping of the ventral mandibular bone thickness revealed pronounced bony mandibular protrusions in animals in group B with obvious bone thinning apically to the left mandibular 4th premolar tooth and left mandibular first and second molar tooth apices (Figure 2). Two degus were randomly chosen from both groups. Mandibular bone volume and thickness located apically to the 4th premolar tooth were significantly smaller/thinner in group B in comparison with group A (*p* < 0.0001). Also, significant differences were recorded (*p* < 0.0001) when comparing the mandibular cortical volume and thickness apically to the left mandibular first and second molar teeth n between the groups. The thinnest bone was detected in group B (0.004 mm), where the left mandibular 4th premolar tooth almost perforated mandibular cortex (Table 1).

**Table 1:** Volume micro-CT measurement of mandibular cortical bone thickness and volume at the area beneath the premolar or molar apex in degus fed by normal (group A) and high phosphorus (group B).

**Tooth**	**Median**	**SD**	**Minimal value**	**Maximal value**	**No. of measurements**
**Mandibular cortical bone thickness (in mm)**
Group A
Degu 1
P4	0.32	0.19	0.01	0.93	1,228
M1	0.35	0.22	0.01	0.90	1,380
M2	0.47	0.2	0.01	1.0	1,821
Degu 2
P4	0.25	0.14	0.02	0.55	932
M1	0.30	0.17	0.02	0.96	1,086
M2	0.45	0.25	0.02	0.98	1,725
Group B
Degu 3
P4	0.19	0.11	0.01	0.59	1,045
M1	0.09	0.05	0.01	0.18	305
M2	0.33	0.18	0.01	0.69	1,226
Degu 4
P4	0.33	0.20	0.004	0.94	1,299
M1	0.22	0.15	0.01	0.81	833
M2	0.37	0.23	0.01	1.0	1,453
**Mandibular cortical bone volume (in** **μm**^**3**^**)**
Group A
Degu 1
P4	906,769.0	1,567,448.67	359.49	12,672,575.07	1,228
M1	514,200.3	2,203,466.41	644.8553201	29,568,676.1	1,380
M2	2,032,739.3	1,717,204.0	676.20	13,128,047.03	1,821
Degu 2
P4	1,335,231.8	939,582.78	3,320.98	5,823,803.71	932
M1	520,842.9	3,502,757.92	2,388.87	25,046,604.20	1,086
M2	2,187,901.8	1,732,968.76	3,870.21	11,698,095.90	1,725
Group B
Degu 3
P4	504,544.3	464,794.42	131.02	3,603,635.30	1,045
M1	350,871.6	1,510,813.11	4,699.68	10,903,065.93	305
M2	1,490,639.5	1,585,699.27	9,952.71	10,622,917.06	1,226
Degu 4
P4	779,456.7	1,466,840.05	311.76	10,608,606.73	1,299
M1	646,052.2	1,451,301.22	1,721.43	11,977,395.76	833
M2	711,809.7	3,545,782.91	547.71	1,9387,953.02	1,453

*P4—4th premolar tooth; M1—1st molar tooth; M2—2nd molar tooth*.

## Discussion

The aim of the present investigation was to examine the chronic effect of high-phosphorus diet on the mandibular apical alveolar bone thickness in a hystricomorph rodent—*Octodon degu*—using microcomputed tomography in four randomly selected animals (two animals from each group). General overview and mapping of the ventral mandibular bone thickness revealed pronounced bony mandibular protrusions in animals fed a high-phosphorus diet with obvious bone thinning apically to the left mandibular 4th premolar tooth and first and second molar tooth apices. Mandibular bone volume and thickness located apically to the 4th premolar tooth and first and second molar teeth were statistically significantly smaller/thinner in group fed by high phosphorus diet.

Degus were fed by a different diet with a calcium-to-phosphorus ratio of 2.1:1 and 0.96:1 from the age of 12 weeks. Degus reach sexual maturity and reach their adult body weight at the age of 12–16 weeks and 5–6 months, respectively (22). High dietary phosphate intake reduced growth, skeletal material, and structural properties and decreased bone strength also in growing or adult rats and mice (23–25), even with adequate amount of calcium as in the present study. As the 4th premolar and molar teeth in degus erupt continuously, the effect of mineral metabolism can affect the teeth in any age. The previous articles showed that teeth of a degu, a hystricomorph rodent with continuously erupting incisors, premolar teeth, and molar teeth, under a high-phosphorus diet elongated coronally and apically had impaired enamel and dentin apposition, and these degus had also decalcified jaws (17–19, 21). The present study showed that degus chronically fed by a high-phosphorus diet had significantly thinner ventral mandibular cortical thickness when compared with degus fed by normal diet. The thinnest cortical/alveolar bone in the group fed by a high-phosphorus diet measured as less as 0.004 mm, where the apex of the mandibular 4th premolar almost perforated the ventral mandibular cortex. As the reserve crowns become elongated, the tooth migrates slightly in the alveolar socket and their curvature is changing and becomes bended (26). Therefore, cortical bone thickness and volume at the apical tooth area can be even little larger in affected animals as in chinchillas (26).

Similar studies of metabolic bone disease and its influence on alveolar bone were also published in rats and mice (27, 28); however, the influence of different environmental, infectious, or metabolic factors on the growing tooth, alveolar bone formation, and other bone pathologies must be done experimentally on growing animals, e.g., very young rats and mice. In contrast, degus have continuously growing dentition, and the effect of any of the above listed factors can be studied in this animal model at any age and for longer time periods. Chronicity of the induced negative or positive factors can be studied as well. Nevertheless, further studies are needed with evaluation of all subjects in the study to include female degus to prove the hypothesis.

Although phosphorus is an essential nutrient, in excess it could be linked to tissue damage by a variety of mechanisms involved in the endocrine regulation of extracellular phosphate, specifically the secretion and action of fibroblast growth factor 23 and parathyroid hormone. Disordered regulation of these hormones by high dietary phosphorus may be the key factor contributing to renal failure, cardiovascular disease, and osteoporosis (29, 30). It is why the authors propose a degu as an animal model of phosphate metabolism disturbances.

The assessment of alveolar bone thickness in previous studies usually relied on dental casts or two-dimensional anteroposterior radiographs (31, 32). Evaluation on two-dimensional anteroposterior radiographs had, however, shortcomings of magnification, geometric distortion, superimposed structures, and inconsistent head position (33, 34). Compared with traditional methods, cone-beam computed tomography (CBCT) could overcome these shortcomings and measure the inclination as well as alveolar bone thickness of teeth with great accuracy on different levels and dimensions (33, 34). CT is nowadays a widely used technique used for the evaluation of normal bone thickness of various anatomical structures, for bone pathology diagnostics and surgical planning (35, 36). In case of dentistry, CT cortical bone thickness evaluation is utilized in many diagnostic and therapeutic procedures. CT is used in orthodontics for proper orthodontic miniplate placements (37, 38). Recent studies from human and experimental dentistry also showed that cone beam CT is useful for the assessment of buccal and lingual alveolar bone thickness, teeth evaluation, and dentoskeletal changes (34, 39–44).

Surface and volume thickness measurement can be also used in pet animals, especially in case of the treatment planning and prognosis of surgical procedures such as tooth extractions, and odontogenic abscess management in small herbivorous animals, as in advanced cases there is a risk of mandibular fracture due to alveolar bone lysis.

## Data Availability Statement

The raw data supporting the conclusions of this article will be made available by the authors, without undue reservation.

## Ethics Statement

The animal study was reviewed and approved by the Branch Commission for Animal Welfare of the Ministry of Agriculture of the Czech Republic (No. 46613/2003-1020).

## Author Contributions

VJ was a major contributor in the research planning, analyzing and interpreting of the data, and manuscript writing. EJ contributed in literature review, data and statistical analyses, and manuscript writing. AB, TZ, and JK were responsible for micro-CT imaging, data analysis, and manuscript writing. All authors contributed to the article and approved the submitted version.

## Funding

This study was supported by the Czech Science Foundation (GACR 524/08/P564) and CzechNanoLab Research Infrastructure supported by MEYS CR (LM2018110). TZ thanks the Grant Agency of the Czech Republic grant 21-05146S. JK thanks the support of grant FSI-S-20-6353. EJ thanks the grant of the Ministry of Agriculture of Czech Republic (RO0518).

## Conflict of Interest

The authors declare that the research was conducted in the absence of any commercial or financial relationships that could be construed as a potential conflict of interest.

## Publisher's Note

All claims expressed in this article are solely those of the authors and do not necessarily represent those of their affiliated organizations, or those of the publisher, the editors and the reviewers. Any product that may be evaluated in this article, or claim that may be made by its manufacturer, is not guaranteed or endorsed by the publisher.
